# Population Genomics of *Mycobacterium tuberculosis* in Ethiopia Contradicts the Virgin Soil Hypothesis for Human Tuberculosis in Sub-Saharan Africa

**DOI:** 10.1016/j.cub.2015.10.061

**Published:** 2015-12-21

**Authors:** Iñaki Comas, Elena Hailu, Teklu Kiros, Shiferaw Bekele, Wondale Mekonnen, Balako Gumi, Rea Tschopp, Gobena Ameni, R. Glyn Hewinson, Brian D. Robertson, Galo A. Goig, David Stucki, Sebastien Gagneux, Abraham Aseffa, Douglas Young, Stefan Berg

**Affiliations:** 1 Genomics and Health Unit, FISABIO Public Health, Valencia 46020, Spain; 2 CIBER (Centros de Investigación Biomédica en Red) in Epidemiology and Public Health, Instituto de Salud Carlos III, Madrid 28029, Spain; 3 Armauer Hansen Research Institute, PO Box 1005, Addis Ababa, Ethiopia; 4 Epidemiology and Public Health, Swiss Tropical and Public Health Institute, Basel 4002, and University of Basel, Basel 4003, Switzerland; 5 Aklilu Lemma Institute of Pathobiology, Addis Ababa University, PO Box 1176, Addis Ababa, Ethiopia; 6 Bovine TB Research Group, Animal and Plant Health Agency, Surrey KT15 3NB, UK; 7 Center for Molecular Bacteriology and Infection, Department of Medicine, Flowers Building, South Kensington, Imperial College London, London SW7 2AZ, UK; 8 Department of Medical Parasitology and Infection Biology, Swiss Tropical and Public Health Institute, Basel 4002, and University of Basel, Basel 4003, Switzerland; 9 The Francis Crick Institute, Mill Hill Laboratory, The Ridgeway, Mill Hill, London NW7 1AA, UK

## Abstract

Colonial medical reports claimed that tuberculosis (TB) was largely unknown in Africa prior to European contact, providing a “virgin soil” for spread of TB in highly susceptible populations previously unexposed to the disease [1, 2]. This is in direct contrast to recent phylogenetic models which support an African origin for TB [3, 4, 5, 6]. To address this apparent contradiction, we performed a broad genomic sampling of *Mycobacterium tuberculosis* in Ethiopia. All members of the *M. tuberculosis* complex (MTBC) arose from clonal expansion of a single common ancestor [7] with a proposed origin in East Africa [3, 4, 8]. Consistent with this proposal, MTBC lineage 7 is almost exclusively found in that region [9, 10, 11]. Although a detailed medical history of Ethiopia supports the view that TB was rare until the 20^th^ century [12], over the last century Ethiopia has become a high-burden TB country [13]. Our results provide further support for an African origin for TB, with some genotypes already present on the continent well before European contact. Phylogenetic analyses reveal a pattern of serial introductions of multiple genotypes into Ethiopia in association with human migration and trade. In place of a “virgin soil” fostering the spread of TB in a previously naive population, we propose that increased TB mortality in Africa was driven by the introduction of European strains of *M. tuberculosis* alongside expansion of selected indigenous strains having biological characteristics that carry a fitness benefit in the urbanized settings of post-colonial Africa.

## Highlights

• Ethiopia hosts a complex population structure of *Mycobacterium tuberculosis*
• Our analyses provide further support for an African origin for tuberculosis
• Our analyses discard the “virgin soil” hypothesis of tuberculosis in Sub-Saharan Africa

## Teaser

Comas et al. highlight a complex population structure of *Mycobacterium tuberculosis* in Ethiopia through genome sequencing. Analyses reveal a mixture of sub-lineages with global distribution as well as those specific to Africa, providing further support for an African origin for tuberculosis and contradicting the earlier “virgin soil” hypothesis.

## Results and Discussion

### Population Structure of *M. tuberculosis* in Ethiopia

**Figure 1:**
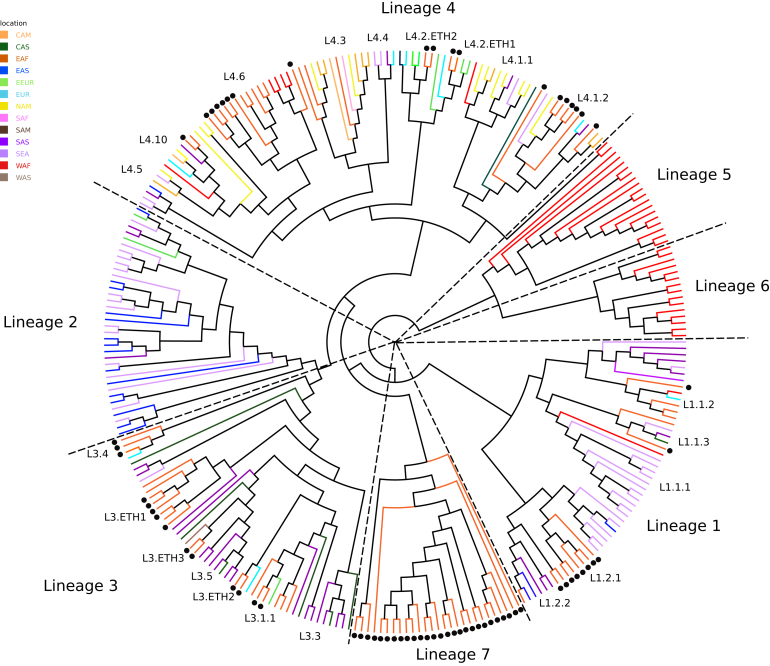
Topology Obtained by Bayesian Analyses as Described in Experimental Procedures Note that no branch length information is used. The topology is highly congruent with the topology from neighbor-joining analysis (see Figure S1). For both analyses, bootstrap values and Bayesian posterior probability values were higher than 95% in almost all nodes. The tree is rooted with *Mycobacterium africanum* strains that are the most basal clades within the *Mycobacterium tuberculosis* complex [3]. External branches are color coded according to the geographic region of the patient from which the isolate was collected. Black dots indicate that the strain was isolated in Ethiopia. The groups identified within each lineage correspond to the groups delineated using a set of diagnostic SNPs as explained in Experimental Procedures. Groups with no diagnostic SNP or that do not form a monophyletic group within the sub-lineage were labeled LX.ETHX. Geographic region: CAM, Central America; CAS, Central Asia; EAF, East Africa; EAS, East Asia; EEUR, Eastern Europe; EUR, Europe; NAM, North America; SAF, South Africa; SAM, South America; SAS, South Asia; SEA, Southeast Asia; WAF, West Africa; WAS, West Asia.

The high incidence of tuberculosis (TB) among Africans during European colonization in the late 19^th^ and early 20^th^ centuries gave rise to the hypothesis that Africa was a “virgin soil,” particularly permissive to the spread of the disease in previously unexposed populations [1, 2]. This stands in sharp contrast to more recent evolutionary models that propose an African origin for *Mycobacterium tuberculosis* [3, 4, 5]. To address the apparent contradiction between an African origin of human TB and the “virgin soil” hypothesis, we performed a broad genomic sampling of *M. tuberculosis* in Ethiopia. We sequenced 66 strains from a previously genotyped *M. tuberculosis* collection [9], selected to represent the most common spoligotypes found in Ethiopia along with examples of rare genotypic outliers and high-density sampling of the unique Ethiopian lineage 7 (L7) (Table S1). Figure 1 illustrates the phylogenetic structure of these Ethiopian isolates in the context of a previously described panel of 219 *M. tuberculosis* complex (MTBC) strains representative of the global diversity [3]. In addition to L7, the Ethiopian isolates belonged to three *M. tuberculosis* lineages: lineage 1 (L1), commonly associated with populations living around the Indian Ocean; lineage 3 (L3), common in Central Asia but also prevalent in East Africa; and lineage 4 (L4), the widespread Euro-American lineage [14]. Mapping to a recent SNP-based classification system [15] identified representatives of five of the eight L4 sub-lineages in the Ethiopian genome dataset. Within L3, we were able to assign Ethiopian strains to three of the five defined sub-lineages along with novel sub-lineages that we provisionally name as L3.ETH1, L3.ETH2, and L3.ETH3. Within L1, we assigned Ethiopian strains to four of the eight defined sub-lineages.

**Figure 2:**
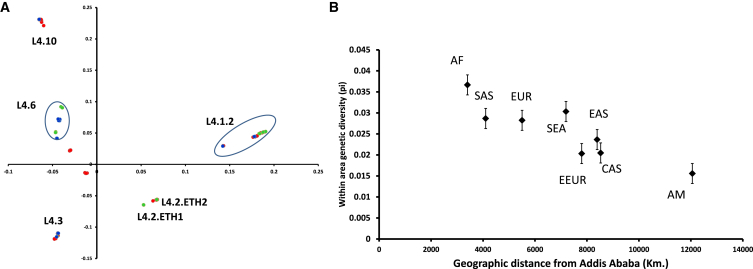
Principal-Component Analysis Using the SNP Matrices Derived from Whole-Genome Analysis (A) The PCA shown is for lineage 4; the corresponding PCA for lineages 3 and 1 are shown in Figures S2 and S3, respectively. The colors represent strains with known African origin (blue) or Eurasian and American origin (red) from a global reference collection and strains with Ethiopian origin (green). (B) The correlation between genetic diversity within a geographic area and the geographic distance from Addis Ababa. Error bars indicate the variance in diversity indices within a region. Geographic region: AF, Africa; SAS, South Asia; EUR, Europe; SEA, Southeast Asia; EEUR, Eastern Europe; EAS, East Asia; CAS, Central Asia; AM, America.

We then explored whether the sub-lineages observed in Ethiopia had a likely African origin by comparing them with our global reference dataset [3]. By combining principal-component analysis (PCA) and the sub-lineage classification described above, we classified the groups assigned to the Ethiopian strains as being of likely “African” or “non-African” origin. Figure 2A shows a PCA of the L4 sub-lineages color coded according to their most likely geographic origin. The classic description of L4 as the “Euro-American” lineage is reflected in the high percentage of non-African strains in Figure 2A, though L4 strains are in fact geographically widespread, including genotypes such as sub-lineage L4.6 that are found only in Africa. Within the more cosmopolitan L4 sub-lineages, Ethiopian L4.2 strains cluster with Eurasian strains belonging to the Ural family at two branchpoints in Figure 1, suggesting their introduction into Ethiopia from Central Asia or from some common ancestral homeland [16]. The coalescent point for sub-lineage 4.2 indicates more recent dissemination as compared to the deep-rooted African sub-lineage L4.6 (Figure 1). Similarly, deep-rooted African strains can be found within L3 and L1 (Figures S2 and S3). L3.ETH1 is comprised exclusively of Ethiopian isolates, for example, and deep-rooted L1.2.1 suggests an early introduction into Ethiopia.

In summary, we find strains belonging to sub-lineages with a global distribution as well as strains from sub-lineages specific to Africa or even to Ethiopia. This diversity of *M. tuberculosis* mirrors the complex admixture of African and non-African haplotypes revealed by analysis of human genetic diversity in Ethiopian populations [17, 18].

### The Horn of Africa as the Likely Place of Origin of TB

**Figure 3:**
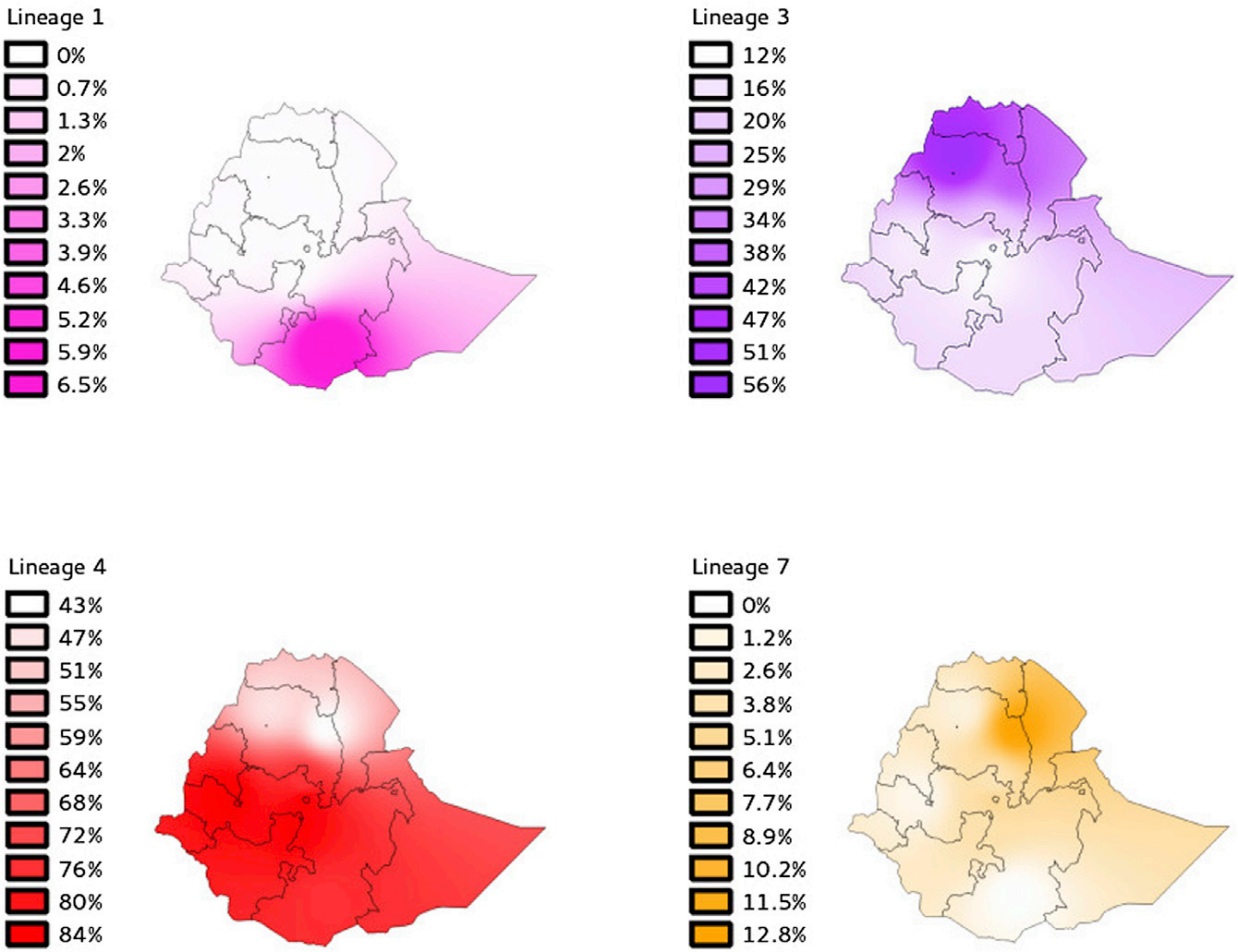
Contour Maps Derived from Point Estimation of the Frequency of Each Lineage in the Different Sampling Locations The results are based on a previously published *M. tuberculosis* collection [9]. Note that there are different scales for each lineage, reflecting their maximum and minimum frequency across the country.

By integrating genome data with our previously reported spoligotype frequencies and collection sites [9], we built contour maps representing the geographic distribution of the principal *M. tuberculosis* genotypes across Ethiopia (Figure 3). L4 dominates across the entire country, L3 is widespread but more frequent in the north, and L1 occurs only in southern Ethiopia. L7 is largely restricted to the highlands around Woldiya and Gondar in northern Ethiopia. The prevalence of L3 in northern Ethiopia is mirrored by the predominant SIT25 spoligotype (L3.ETH1) in neighboring Sudan [19]. Similarly in the south, L1 strains are common in neighboring Somalia [10] (Figure S4). The geographic restriction of L7 could have arisen if the infection was maintained within a stable and isolated human population. Alternatively, it is possible that strains from this lineage have acquired mutations that enhance their infection and transmission in the context of some particular host genetic background but incur a fitness cost in other populations. Similar patterns have been observed in other *M. tuberculosis* populations [20] and in *Helicobacter pylori* [21].

Placing the Ethiopian strains in the context of the global diversity dataset, we plotted within-area genetic diversity against the geographic distances from a midpoint in the African continent and found a strong negative correlation (r = −0.81, p < 0.05) (Figure 2B). When we repeated the calculation specifying West Africa as the starting point of diversification, we found a similar correlation (r = −0.79, p < 0.05). However, when we specified the coordinates of Addis Ababa—the capital of Ethiopia—as the point of origin, the correlation became stronger and with higher statistical significance (r = −0.84, p < 0.01), pointing to East Africa as the likely place of origin of the MTBC. Taken together, our results indicate that the geographic distance to Africa can explain up to 71% of the global genetic diversity of the human-adapted MTBC, which is consistent with serial bottlenecks following initial emergence from a genetically diverse pool in Africa [5]. The strong parallels with patterns identified in human populations [22] and for pathogens such as *Plasmodium falciparum* and *Helicobacter pylori* [23, 24] are striking but consistent with epidemiological observations of robust associations between MTBC genotypes with human populations despite frequent redistribution following increased globalization [20].

### Molecular Dating Contradicts the “Virgin Soil” Hypothesis

**Figure 4:**
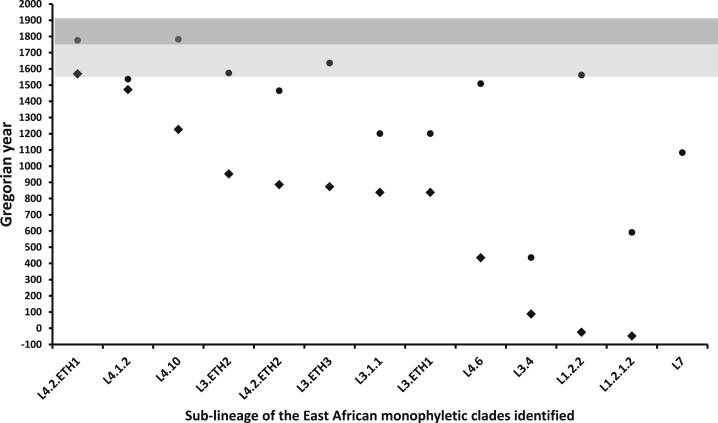
Representation of Dating Events for MTBC Sub-lineages Included in This Study Dots (•) represents the age of the most recent common ancestor of the different Ethiopian sub-lineages circulating today. Diamonds (♦) represent the split of those Ethiopian groups from the closest non-Ethiopian strains in the global dataset described in [3]. Note that for lineage 7, the split from other MTBC was the third millennium BCE and is not represented. Light gray highlights the time of first sporadic European contacts in Ethiopia (16^th^–19^th^ centuries CE). Dark gray highlights the time frame for a more continuous contact between Ethiopia and foreign nations (19^th^ century CE and onward). The data presented show only the results for the MTBC-6 model in which the whole complex is predicted to be around 6,000 years old [25]; the results for the MTBC-70 model where all lineages were already established by 4,000 years ago are not shown. (See Table S2.)

To review the “virgin soil” hypothesis in the context of an African origin for TB, we tested whether the MTBC lineages and sub-lineages currently circulating in Ethiopia appeared before or after Ethiopia experienced major contacts with Europeans. Although Ethiopia escaped colonization sensu stricto, human genetic diversity data as well as historical records indicate that the region was a bridge between Africa and Eurasia [17, 18]. We analyzed the Ethiopian MTBC genomes in the context of our global diversity dataset and the two competing models for the timing of early events in the evolution of the MTBC. We identified two coalescent points for each of the clusters containing Ethiopian isolates, corresponding to (1) the point of origin of the common ancestor of the cluster and (2) the point at which Ethiopian genotypes diverged from genetically related strains outside of East Africa. We performed BEAST analysis using the 70-thousand-year time frame (MTBC-70 [3]) and the 6-thousand-year time frame (MTBC-6 [25]). The predicted coalescent times obtained using the two models are shown for the major Ethiopian genotypes in Table S2, while Figure 4 shows the estimated coalescent times for the MTBC-6 model.

We were unable to determine which of the two competing models was more likely in the face of historical or pre-historical records (although see Supplemental Experimental Procedures for an extended discussion of this topic). However, both models are clearly incompatible with the view that TB did not exist in Ethiopia or in Sub-Saharan Africa prior to European contact as envisaged in the “virgin soil” hypothesis. Specifically, MTBC-70 dating suggests that the main lineages and sub-lineages were already established 4,000 years ago or earlier (Table S2). The coalescent point for entry of the dominant Ethiopian L4.2.ETH1 cluster is consistent with its arrival in association with the major 3,000-years-ago north-south human migration and origin of the Ethiosemitic languages (95% highest posterior density [HPD] interval: 2,055–3,835 years ago for the divergence within Ethiopia) [26]. Other sub-lineages were already established 5,000 years ago and may be linked to the recent discovery of significant Eurasian admixture in Ethiopia, explained by back migrations of earlier Neolithic farmers probably from Anatolia around that time [18]. In contrast, the MTBC-6 model suggests that some sub-lineages like L4.2.ETH1 (95% HPD interval: 167–313 years ago) or L4.1.2 (95% HPD interval: 404–539 years ago) could have been introduced at the time of early Portuguese contact with Ethiopia in the 16^th^ century or later, while other sub-lineages are several centuries older (Figure 4). In agreement with the PCA plots (Figures 2A, S3, and S4), MTBC-6 predicts that some sub-lineages of lineage 1, lineage 3, and lineage 4 were present in East Africa prior to the 10^th^ century (Table S2; Figure 4), long before the onset of European colonization. The most extreme case of this pattern is L7, with the MTBC-70 time frame suggesting that L7 was branching off around the time of initial human migrations out of Africa (Table S2). MTBC-6 predicts a common ancestor reaching back to the 11^th^ century (95% HPD interval: 806–1,191 years ago) and divergence of L7 from related MTBC strains as early as the third millennium BCE (95% HPD interval: 4,099–4,850 years ago; Table S2; Figure 4). The dating analyses reveal that the same observation of pre-colonial presence of lineages also applies to other parts of Africa. For example, the West Africa-restricted lineages 5 and 6 (L5 and L6), also known as *Mycobacterium africanum*, are predicted to have a common ancestor 5,000 years ago under the MTBC-6 model. In summary, both dating models are incompatible with the “virgin soil” hypothesis for Sub-Saharan Africa.

### Conclusions

Understanding the factors underlying the historical persistence or replacement of particular sub-populations of *M. tuberculosis* has potential relevance for predicting future trends in disease epidemiology. There is current evidence that strains belonging to ancient African lineages L5 and L6 are slowly being replaced by L4 in West Africa, and that L2 strains (prevalent in East Asia) are expanding in South Africa (reviewed in [14]), for example, and there is an urgent need to identify the mechanisms that determine expansion or restriction of emerging drug-resistant genotypes. Successful introduction of new genotypes could be driven by major population migrations or by fitness properties linked to host genotype or social environment [27]. A simple conceptual model to account for the population structure in Ethiopia would envisage that prolonged stable co-evolution of human and microbial populations favors a relatively benign infection that optimizes mutual survival of both partners, whereas an unstable environment favors expansion of more aggressive microbial genotypes irrespective of their long-term impact on host populations. TB epidemiology in colonial Africa would then reflect replacement of a stable *M. tuberculosis* population with introduction of aggressive genotypes selected in industrialized Europe alongside expansion of opportunist indigenous genotypes with the ability to exploit opportunities of African urbanization [28]. This can explain the high heterogeneity in infection rates across Sub-Saharan Africa observed after colonial contact [29]. Consistent with this model, the most recently introduced sub-lineage L4.1.2 is associated with large transmission clusters in Ethiopia [9]; an independent genotyping study in the Amhara region similarly demonstrated higher transmission and drug resistance associated with spoligotypes corresponding to L4.1.2 (Haarlem) and L4.2.ETH1 (named NW-ETH3 in [30, 31]). In contrast, patients infected with L7 strains appear to report later to the health clinics and tend not to be associated with recent transmission and multidrug resistance [30].

## Experimental Procedures

### Preparation of Genomic DNA and Genome Sequencing

All Ethiopian samples were obtained from a previous study [9]. Selected MTBC strains were cultured from frozen stocks on Middlebrook 7H11 agar. One single colony per strain was then sub-cultured, cells were harvested by heat inactivation, and genomic DNA was purified using a standard protocol [32] and utilized for sequencing on an Illumina HiSeq platform at GATC Biotech (Konstanz, Germany) with coverage ranging between 150 and 244 reads per base pair. Reads were mapped to a reconstructed most recent common ancestor of the MTBC as described previously [33]. For each strain, a combination of BWA mapping and SAMtools SNP calling [34] was used with the parameters set as described earlier [9]. We controlled for false positives by removing calls falling in repetitive regions [33]. SNP calls should have been present in at least 10 reads, with SNP mapping qualities of the bases higher than 20. The 66 sequenced genomes were combined with a recently published whole-genome dataset of 219 global MTBC isolates [3].

### Statistical Analyses

To define the population structure of *M. tuberculosis* in Ethiopia, we scanned the strains for diagnostic SNPs defining phylogenetic groups as recently published [15]. To associate the different Ethiopian phylogenetic groups with global African or non-African clades, we carried out PCA using Stata [35]. To explore the possible relationship between genetic diversity and geographic distance, we classified MTBC strains into 13 broad geographic areas. These areas are approximately the same as those defined by the United Nations [3]. The within-area genetic diversity (pi) was calculated using DnaSP [20]. We used the Pearson correlation coefficient to test for correlation between pi for MTBC and the mean geographic distance between the country of origin of the patient and an arbitrary point representing (1) a midpoint in Africa (latitude 6.793981° N, longitude 21.299250° E), (2) Addis Ababa (8.984711° N, 38.754112° E), or (3) West Africa (12.433722° N, 1.102581° W).

Contour maps to infer the frequency of each lineage across the country were built using available genotyping data of almost 1,000 *M. tuberculosis* isolates previously generated [9]. We inferred the incidence of the lineage across the country using the inverse distance weighting interpolation with a power value of 2.7. All analyses were performed using QGIS v2.8, which is freely available at http://www.qgis.org/en/site/.

### Phylogenetic and Dating Analyses

For phylogenetic inference, we used a core genome alignment of SNPs after removing columns with gaps (deletions or heterozygous calls). Phylogenetic analyses were performed using MEGA for the neighbor-joining (NJ) tree (Tamura-3 parameters) with 1,000 bootstrap pseudo-replicates [36]. RAxML version 8.0.0 [37] was used for the maximum-likelihood approach specifying a general time-reversible model of nucleotide evolution and estimating five gamma categories and 1,000 bootstrap pseudo-replicates. BEAST v1.7.5 was used for the Bayesian topology inference as specified below [38]. The application of different inference methods or models of nucleotide evolution did not have an impact on the phylogeny, with all relevant clades discussed in this manuscript having a support value of 100 for the three inference methods (see Figure 1 for the maximum-likelihood topology and Figure S1 for details of support values of the main clades discussed).

The dating analyses were performed using similar parameters as in earlier publications [3, 25]. Briefly, the phylogenetic tree was calibrated with the two existing competing models for predicting the origin of the MTBC: a 70,000-year-old ancestor [3] or a 6,000-year-old ancestor [25] was pre-specified in BEAST. We used an uncorrelated log-normal distribution to model the variation of the substitution rate among branches, a Hasegawa-Kishino-Yano nucleotide substitution model, and a skyline demographic model with ten different intervals. We ran between six and ten chains during 5 × 10^7^ generations and sampled every 1 × 10^4^ generations to assure independent convergence. The burn-in was set to 20%. Convergence was evaluated in Tracer [39] with all relevant parameters reaching an effective sample size > 100.

## Author Contributions

Study design was performed by S. Berg, I.C., S.G., D.Y., A.A., E.H., R.G.H., B.D.R., R.T., B.G., and G.A. Data collection (sampling, mycobacterial culturing, molecular typing) was performed by T.K., E.H., S. Bekele, W.M., B.G., I.C., and S. Berg. Data analyses were performed by I.C., S. Berg, D.Y., S.G., G.A.G., D.S., and A.A. The manuscript was written primarily by I.C., S. Berg, D.Y., S.G., and A.A. All authors read the manuscript and approved its publication. I.C. and S. Berg contributed equally and jointly directed the work.

## Accession Numbers

Raw FASTQ files have been deposited at the EBI Sequence Read Archive under accession numbers SRA: PRJEB9201, PRJEB3163, PRJEB3124.

## Supplemental Information

Document S1. Figures S1–S4, Tables S1 and S2, and Supplemental Experimental Procedures

Document S2. Article plus Supplemental Information
