# Supplementary material for: Population Genomics of Mycobacterium tuberculosis in Ethiopia Contradicts the Virgin Soil Hypothesis for Human Tuberculosis in Sub-Saharan Africa
Source: Curr Biol. 2015 Dec 21;25(24):3260–6. doi: 10.1016/j.cub.2015.10.061 (PMC4691238; doi:10.1016/j.cub.2015.10.061)
Supplement: Document S1. Figures S1–S4, Tables S1 and S2, and Supplemental Experimental Procedures [file mmc1.pdf]

Current Biology

Supplemental Information

Population Genomics of *Mycobacterium tuberculosis* in Ethiopia Contradicts the Virgin Soil Hypothesis for Human Tuberculosis in Sub-Saharan Africa

Iñaki Comas, Elena Hailu, Teklu Kiros, Shiferaw Bekele, Wondale Mekonnen, Balako Gumi, Rea Tschopp, Gobena Ameni, R. Glyn Hewinson, Brian D. Robertson, Galo A. Goig, David Stucki, Sebastien Gagneux, Abraham Aseffa, Douglas Young, and Stefan Berg

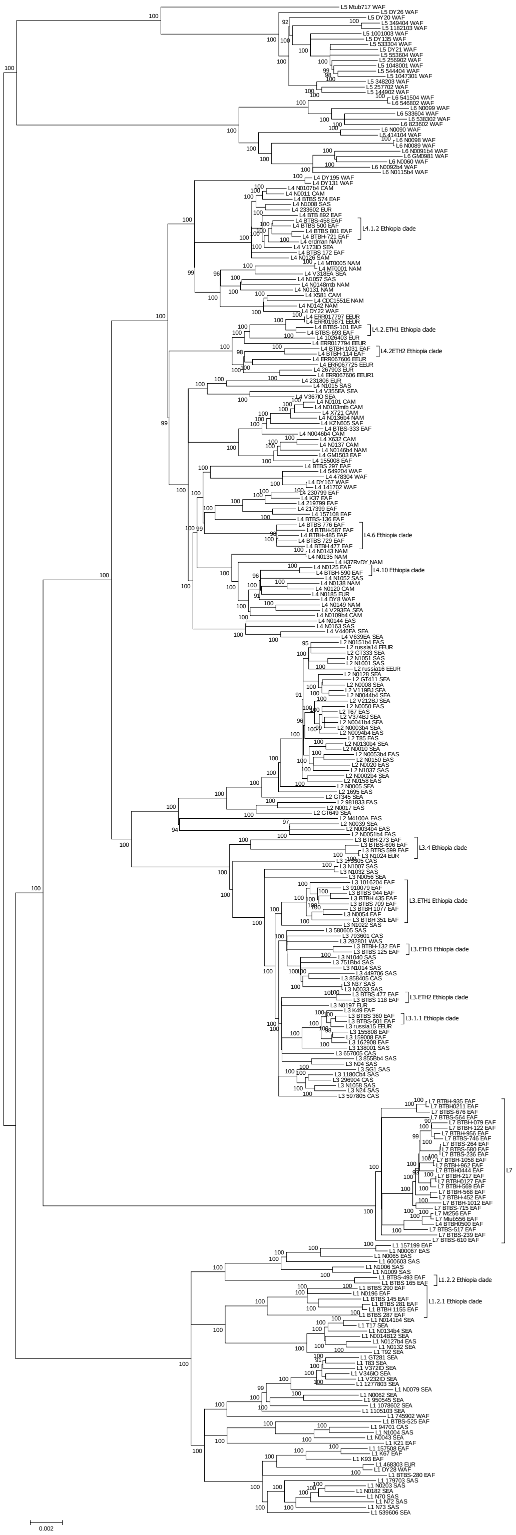

Ethiopian clade	NJ	BS	ML
L4.1.2	100	1	100
L4.2.ETH2	100	1	100
L4.2.ETH1	100	1	100
L4.10	100	1	100
L4.6	100	1	100
L3.1.1	100	1	100
L3.4	100	1	100
L3.ETH3	100	1	100
L3.ETH2	100	1	100
L3.ETH1	100	1	100
L1.2.1	100	1	100
L1.2.2	100	1	100
L7	100	1	100

Figure S1. Maximum Likelihood phylogeny under the General-Time-Reversible model of nucleotide evolution (five gamma categories; 1,000 bootstrap pseudo-replicates). The scale is proportional to the number of substitutions per polymorphic site. Similar topologies were obtained using Neighbour-Joining and Bayesian phylogenetic inference approaches. Insert table shows the support values for the three approaches of the main clades discussed in the text (NJ, Neigbour-Joining bootstrap; BS, Bayesian Support; ML, Maximum-Likelihood bootstrap). Related to Figure 1 and Figure 4.

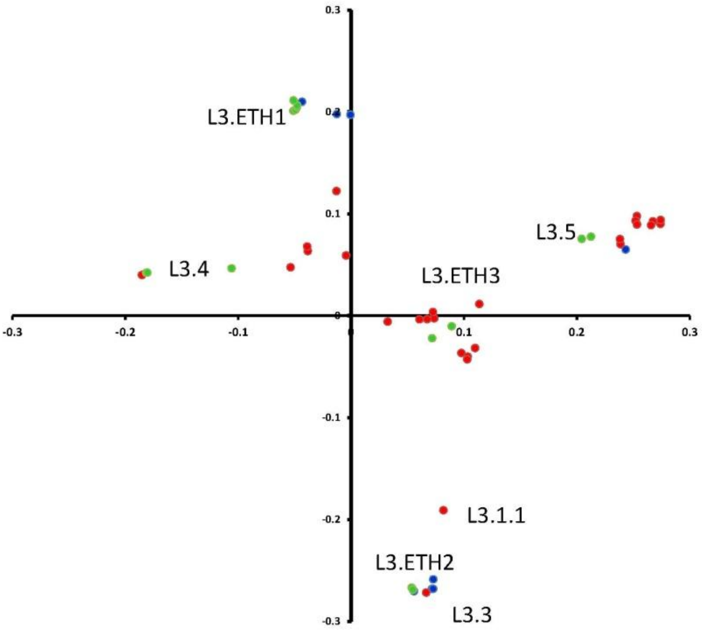

Figure S2. Principal component analysis for the Lineage 3 sub-lineages present in Ethiopia in relation with a global reference strain. The colors represent strains with known African origin (blue), Ethiopian origin (green), or Eurasian and American origin (red). Related to Figure 2.

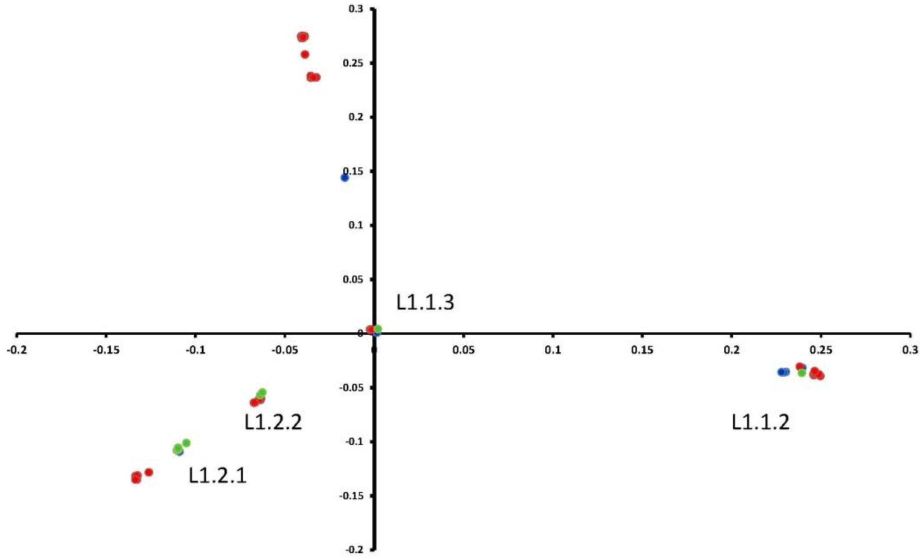

Figure S3. Principal component analysis for the Lineage 1 sub-lineages present in Ethiopia in relation with a global reference strain. The colors represent strains with known African origin (blue), Ethiopian origin (green), or Eurasian and American origin (red). Related to Figure 2.

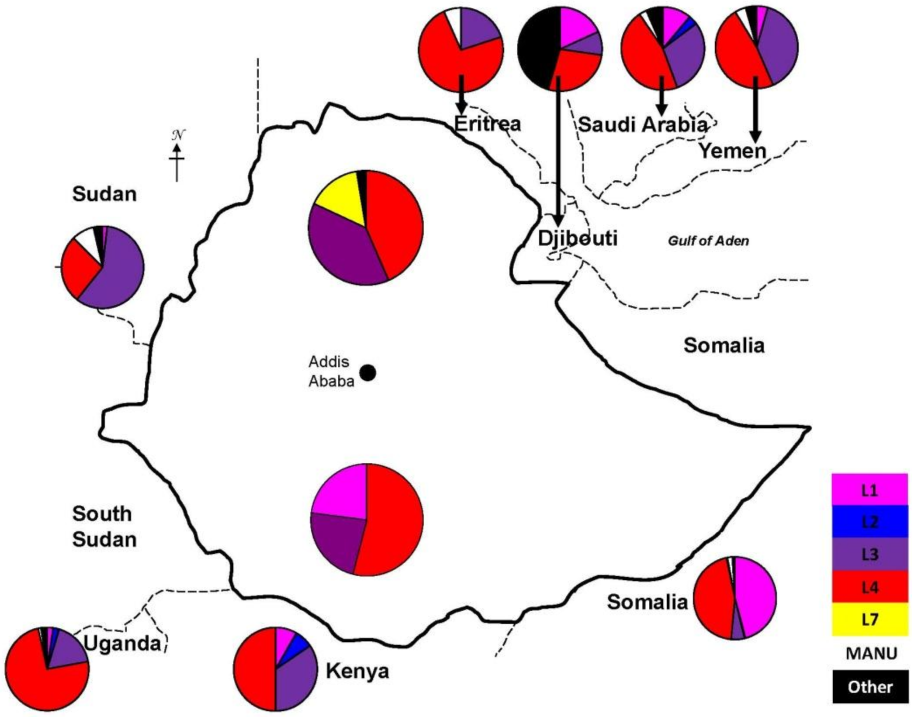

Figure S4. Geographic distribution of main MTBC lineages in the North and the South of Ethiopia based on genotyping of nearly 1000 MTBC isolates [S1]. Corresponding frequencies of MTBC lineages in the neighboring countries have been estimated based on spoligotype data of MTBC isolates registered at the SITVIT database [S2]. The records extracted from this database may not reflect the true prevalence of different lineages in these countries. “MANU” refers to the MANU family [S2] while “Others” refers to isolates of *Mycobacterium canettii* or unknown MTBC lineage. Related to Figure 3.

Table S1. Summary of information of *Mycobacterium tuberculosis* strains from Ethiopia analysed by genome sequencing in this study. Related to Figure 1.

Lineage	Classification	Strain ID	Coverage	Disease type	Collection site	Patient ethnicity	Spoligotype	Spoligotype pattern (filled cell = spacer present; empty cell = spacer absent)																																										
SNP based	Coll et al. 2015		mean				SITVIT2 DB	1	2	3	4	5	6	7	8	9	10	11	12	13	14	15	16	17	18	19	20	21	22	23	24	25	26	27	28	29	30	31	32	33	34	35	36	37	38	39	40	41	42	43
L1	L1.2.1	BTBS0145	243.85	Pulmonary TB	Negelle	Oromo	SIT 43	■	■	■	■	■	■	■	■	■	■	■	■	■	■	■	■	■	■	■	■	■	■			■	■	■	■					■		■	■	■	■	■	■	■	■	■
L1	L1.2.2	BTBS0165	196.146	Pulmonary TB	Negelle	Oromo	SIT 48	■	■	■	■	■	■	■	■	■	■	■	■	■	■	■	■	■	■	■	■	■	■	■	■	■	■	■	■					■		■	■	■	■	■		■	■	■
L1	L1.1.2	BTBS0280	235.585	Pulmonary TB	Filtu	Somali	SIT 10	■			■	■	■	■	■	■	■	■	■	■	■	■	■	■	■		■		■	■	■	■	■	■	■					■		■	■	■	■	■	■	■	■	■
L1	L1.2.1	BTBS0281	233.046	Pulmonary TB	Filtu	Somali	SIT 204	■	■	■		■	■	■	■	■	■	■	■	■	■	■	■	■	■	■	■	■	■	■	■	■	■	■	■					■		■	■	■	■	■	■	■	■	■
L1	L1.2.1	BTBS0287	233.598	Pulmonary TB	Negelle	Oromo	SIT 591	■	■	■	■	■	■	■	■	■	■	■	■	■	■	■	■	■	■	■	■	■	■		■	■	■	■	■					■		■	■	■	■	■	■	■	■	■
L1	L1.2.1	BTBS0290	215.48	Pulmonary TB	Negelle	Oromo	SIT 3332	■	■	■	■	■	■	■	■	■	■	■	■		■	■	■	■	■	■	■	■	■						■					■		■		■	■	■	■	■	■	■
L1	L1.2.2	BTBS0493	237.682	Pulmonary TB	Negelle	Oromo	SIT 48	■	■	■	■	■	■	■	■	■	■	■	■	■	■	■	■	■	■	■	■	■	■	■	■	■	■	■	■					■		■	■	■	■	■		■	■	■
L1	L1.1.3	BTBS0525	235.608	Pulmonary TB	Negelle	Oromo	SIT 924	■	■	■	■	■	■	■	■	■	■	■														■	■	■	■					■		■	■		■	■	■	■	■	■
L1	L1.2.1	BTBH1155	239.488	TB lymphadenitis	Filtu	Somali	SIT 3335	■	■	■		■	■	■	■	■	■	■	■	■	■	■	■	■	■	■	■	■	■			■	■	■	■					■		■	■	■	■	■	■	■	■	■
L3	L3.ETH2	BTBS0118	196.109	Pulmonary TB	Fiche	Oromo	SIT 26	■	■	■					■	■	■	■	■	■	■	■	■	■	■	■	■	■	■													■	■	■	■	■	■	■	■	■
L3	L3.ETH3	BTBS0125	232.262	Pulmonary TB	Negelle	Oromo	SIT 247	■	■	■					■	■	■	■	■	■	■	■	■	■	■	■	■	■	■													■	■	■			■	■	■	■
L3	L3.ETH3	BTBH0132	212.985	TB lymphadenitis	Woldiya	Amhara	Orphan	■							■	■	■	■	■	■	■	■	■	■	■	■	■	■	■																					
L3	L3.4	BTBH0273	236.887	TB lymphadenitis	Woldiya	Amhara	SIT 1200	■	■	■					■	■	■	■	■	■	■	■	■	■	■	■	■	■	■			■	■	■	■	■	■	■	■	■	■	■	■	■	■	■	■	■	■	■
L3	L3.ETH1	BTBH0351	236.904	TB lymphadenitis	Woldiya	Amhara	SIT 25	■	■	■					■	■	■	■	■	■	■	■	■	■	■	■	■	■	■													■	■			■	■	■	■	■
L3	L3.1.1	BTBS0360	223.748	Pulmonary TB	Gondar	Oromo	SIT 21	■	■	■					■	■		■	■	■	■	■	■	■	■	■																	■	■	■	■	■	■	■	■
L3	L3.ETH1	BTBH0435	228.314	TB lymphadenitis	Butajira	Gurage	SIT 25	■	■	■					■	■	■	■	■	■	■	■	■	■	■	■	■	■	■													■	■			■	■	■	■	■
L3	L3.ETH2	BTBS0477	229.68	Pulmonary TB	Negelle	Oromo	SIT 26	■	■	■					■	■	■	■	■	■	■	■	■	■	■	■	■	■	■													■	■	■	■	■	■	■	■	■
L3	L3.1.1	BTBS0501	231.567	Pulmonary TB	Negelle	Oromo	SIT 21	■	■	■					■	■		■	■	■	■	■	■	■	■	■																	■	■	■	■	■	■	■	■
L3	L3.4	BTBS0599	230.005	Pulmonary TB	Butajira	Amhara	SIT 1																																			■	■	■	■	■	■	■	■	■
L3	L3.4	BTBS0696	216.589	Pulmonary TB	Negelle	Amhara	SIT 1																																			■	■	■	■	■	■	■	■	■
L3	L3.ETH1	BTBS0709	244.302	Pulmonary TB	Fiche	Amhara	SIT 25	■	■	■					■	■	■	■	■	■	■	■	■	■	■	■	■	■	■													■	■			■	■	■	■	■
L3	L3.ETH1	BTBS0944	223.858	Pulmonary TB	Gondar	Amhara	SIT 25	■	■	■					■	■	■	■	■	■	■	■	■	■	■	■	■	■	■													■	■			■	■	■	■	■
L3	L3.ETH1	BTBH1077	240.882	TB lymphadenitis	Woldiya	Amhara	SIT 25	■	■	■					■	■	■	■	■	■	■	■	■	■	■	■	■	■	■													■	■			■	■	■	■	■
L4	L4.2.ETH1	BTBS0101	238.678	Pulmonary TB	Gondar	Oromo	SIT 149	■	■	■	■	■	■	■	■	■											■	■	■	■	■	■	■	■	■	■	■	■	■					■	■	■	■	■	■	■
L4	L4.2.ETH2	BTBH0114	237.376	TB lymphadenitis	Butajira	Silti	SIT 3135	■	■	■	■	■	■	■	■	■	■	■	■	■	■	■	■	■	■	■			■	■	■																			
L4	L4.6	BTBS0136	235.904	Pulmonary TB	Filtu	Somali	SIT 584	■	■	■	■	■	■	■	■	■	■	■	■	■	■	■	■		■	■	■	■	■	■	■	■	■	■	■	■	■	■	■					■	■	■		■	■	■
L4	L4.6	BTBS0297	218.856	Pulmonary TB	Negelle	Oromo	SIT 2793	■																		■	■	■	■		■	■	■	■	■	■	■	■	■					■	■	■	■	■	■	■
L4	L4.3	BTBS0333	229.551	Pulmonary TB	Negelle	Oromo	SIT 4																									■	■	■	■	■	■	■	■					■	■	■	■	■	■	■
L4	L4.1.2	BTBS0458	231.67	Pulmonary TB	Gondar	Amhara	SIT 3313																									■							■					■	■			■	■	■
L4	L4.6	BTBH0477	225.319	TB lymphadenitis	Ghimbi	Oromo	SIT 3337	■	■	■	■	■	■	■	■	■	■	■	■		■	■	■	■	■	■	■	■	■	■	■					■	■	■	■					■	■	■	■	■	■	■
L4	L4.6	BTBH0485	235.216	TB lymphadenitis	Ghimbi	Amhara	SIT 3137	■	■	■	■	■	■	■	■		■	■	■		■	■	■	■	■	■	■	■	■	■	■	■	■	■	■	■	■	■	■					■	■	■	■	■	■	■
L4	L4.1.2	BTBS0500	200.73	Pulmonary TB	Negelle	Oromo	SIT 134	■	■	■	■	■	■	■	■	■	■	■	■	■	■	■	■	■	■	■	■	■	■	■	■	■	■	■	■	■	■		■					■	■			■	■	■
L4	L4.1.2	BTBS0574	223.594	Pulmonary TB	Fiche	Oromo	SIT 50	■	■	■	■	■	■	■	■	■	■	■	■	■	■	■	■	■	■	■	■	■	■	■	■	■	■	■	■	■	■		■					■	■	■	■	■	■	■
L4	L4.6	BTBH0587	209.579	TB lymphadenitis	Gondar	Oromo	SIT 37	■	■	■	■	■	■	■	■	■	■	■	■		■	■	■	■	■	■	■	■	■	■	■	■	■	■	■	■	■	■	■					■	■	■	■	■	■	■
L4	L4.10	BTBH0590	209.258	TB lymphadenitis	Ghimbi	Oromo	SIT 53	■	■	■	■	■	■	■	■	■	■	■	■	■	■	■	■	■	■	■	■	■	■	■	■	■	■	■	■	■	■	■	■					■	■	■	■	■	■	■
L4	L4.2.ETH1	BTBS0693	232.159	Pulmonary TB	Negelle	Oromo	SIT 149	■	■	■	■	■	■	■	■	■											■	■	■	■	■	■	■	■	■	■	■	■	■					■	■	■	■	■	■	■
L4	L4.1.2	BTBH0721	225.859	TB lymphadenitis	Butajira	Gurage	SIT 3134	■	■	■	■	■	■	■	■	■	■	■	■		■	■	■	■	■		■	■	■	■	■	■	■	■	■	■	■		■					■	■	■	■	■	■	■
L4	L4.6	BTBS0729	218.798	Pulmonary TB	Holeta	data missing	SIT3314							■	■		■	■	■		■	■	■	■	■	■	■	■	■	■	■	■	■	■	■	■	■	■	■					■	■	■	■	■	■	■
L4	L4.6	BTBS0776	193.269	Pulmonary TB	Ghimbi	Oromo	Orphan	■	■	■	■	■	■	■	■	■	■	■	■		■		■	■	■	■	■	■	■	■	■																			
L4	L4.1.2	BTBS0801	244.19	Pulmonary TB	Butajira	data missing	SIT 3134	■	■	■	■	■	■	■	■	■	■	■	■		■	■	■	■	■		■	■	■	■	■	■	■	■	■	■	■		■					■	■	■	■	■	■	■
L4	L4.2.ETH2	BTBH1031	218.033	TB lymphadenitis	Woldiya	Amhara	SIT 46	■	■	■	■	■	■	■	■	■	■	■	■	■	■	■	■	■	■	■	■	■	■	■	■																			
L7	Not classified	BTBH0079	240.671	TB lymphadenitis	Woldiya	Amhara	SIT 1729	■	■	■																						■					■	■	■	■	■	■	■	■	■	■	■	■	■	■
L7	Not classified	BTBH0122	237.462	TB lymphadenitis	Woldiya	Amhara	SIT 1729	■	■	■																						■					■	■	■	■	■	■	■	■	■	■	■	■	■	■
L7	Not classified	BTBH0127	190.361	TB lymphadenitis	Woldiya	Amhara	SIT 1729	■	■	■																						■					■	■	■	■	■	■	■	■	■	■	■	■	■	■
L7	Not classified	BTBH0211	198.175	TB lymphadenitis	Woldiya	Gurage	SIT 3336	■	■	■																						■	■	■			■	■	■	■	■	■	■	■	■	■	■	■	■	
L7	Not classified	BTBH0217	241.798	TB lymphadenitis	Butajira	Silti	SIT 1729	■	■	■																						■					■	■	■	■	■	■	■	■	■	■	■	■	■	■
L7	Not classified	BTBS0236	235.858	Pulmonary TB	Fiche	Oromo	SIT 1729	■	■	■																						■					■	■	■	■	■	■	■	■	■	■	■	■	■	■
L7	Not classified	BTBS0239	238.313	Pulmonary TB	Fiche	Oromo	SIT 910	■	■	■																						■	■	■			■	■	■	■	■	■	■	■	■	■	■	■	■	■
L7	Not classified	BTBS0264	238.319	Pulmonary TB	Fiche	Oromo	SIT 1729	■	■	■																						■					■	■	■	■	■	■	■	■	■	■	■	■	■	■
L7	Not classified	BTBH0444	185.465	TB lymphadenitis	Woldiya	Amhara	Orphan	■	■	■																						■							■	■	■	■	■	■	■	■	■	■	■	■
L7	Not classified	BTBH0452	190.352	TB lymphadenitis	Woldiya	Tigre	SIT 1729	■	■	■																						■					■	■	■	■	■	■	■	■	■	■	■	■	■	■
L7	Not classified	BTBH0500	150.196	TB lymphadenitis	Woldiya	Amhara	SIT 910	■	■	■																						■	■	■			■	■	■	■	■	■	■	■	■	■	■	■	■	■
L7	Not classified	BTBS0517	232.237	Pulmonary TB	Woldiya	Amhara	SIT 910	■	■	■																						■	■	■			■	■	■	■	■	■	■	■	■	■	■	■	■	■
L7	Not classified	BTBS0564	237.648	Pulmonary TB	Fiche	data missing	SIT 910	■	■	■																						■	■	■			■	■	■	■	■	■	■	■	■	■	■	■	■	■
L7	Not classified	BTBH0568	236.93	TB lymphadenitis	Woldiya	Amhara	SIT 1729	■	■	■																						■					■	■	■	■	■	■	■	■	■	■	■	■	■	■
L7	Not classified	BTBH0569	241.15	TB lymphadenitis	Woldiya	Amhara	SIT 1729	■	■	■																						■					■	■	■	■	■	■	■	■	■	■	■	■	■	■
L7	Not classified	BTBS0580	233.871	Pulmonary TB	Fiche	Oromo	SIT 1729	■	■	■																						■					■	■	■	■	■	■	■	■	■	■	■	■	■	■
L7	Not classified	BTBS0610	238.871	Pulmonary TB	Butajira	data missing	SIT 910	■	■	■																						■	■	■			■	■	■	■	■	■	■	■	■	■	■	■	■	■
L7	Not classified	BTBS0676	235.172	Pulmonary TB	Addis Ababa	data missing	SIT 910	■	■	■																						■	■	■			■	■	■	■	■	■	■	■	■	■	■	■	■	■
L7	Not classified	BTBH0715	228.811	TB lymphadenitis	Fiche	Amhara	Orphan		■	■																						■	■	■			■	■	■	■	■	■	■	■	■	■	■	■	■	■
L7	Not classified	BTBS0746	216.68	Pulmonary TB	Woldiya	Amhara	SIT 1729	■	■	■																						■					■	■	■	■	■	■	■	■	■	■	■	■	■	■
L7	Not classified	BTBH0935	221.315	TB lymphadenitis	Butajira	Gurage	SIT 3336	■	■	■																						■	■	■			■	■	■	■	■	■	■	■	■	■	■	■	■	
L7	Not classified	BTBH0956	210.431	TB lymphadenitis	Woldiya	Amhara	SIT 1729	■	■	■																						■					■	■	■	■	■	■	■	■	■	■	■	■	■	■
L7	Not classified	BTBH0962	228.625	TB lymphadenitis	Woldiya	data missing	SIT 1729	■	■	■																						■					■	■	■	■	■	■	■	■	■	■	■	■	■	■
L7	Not classified	BTBH1012	219.632	TB lymphadenitis	Butajira	data missing	SIT 910	■	■	■																						■	■	■			■	■	■	■	■	■	■	■	■	■	■	■	■	■
L7	Not classified	BTBH1058	238.187	TB lymphadenitis	Woldiya	Amhara	SIT 1729	■	■	■																						■					■	■	■	■	■	■	■	■	■	■	■	■	■	■

Table S2. Coalescent times in MTBC evolution for the most frequent Ethiopian clades identified in this study. Median height values for two coalescent events are shown for respective sub-lineage (see text for details). The dates are given in years before present. Related to Figure 4.

	**MTBC-70 (years ago)**	**MTBC-6 (years ago)**	**MTBC-70 (years ago)**	**MTBC-6 (years ago)**
**Coalescent event**	**Split from closest non-East African**		**Divergence within East Africa**	
**L4.1.2**	6,565	538	5,759	473
**L4.2.ETH2**	13,849	1,124	6,628	545
**L4.2.ETH1**	5,548	441	2,863	234
**L4.10**	9,904	784	2,847	228
**L4.6**	19,669	1,575	6,009	501
**L3.1.1**	14,300	1,172	9,831	809
**L3.4**	23,763	1,922	19,383	1,574
**L3.ETH3**	15,956	1,137	5,201	374
**L3.ETH2**	14,107	1,058	4,392	436
**L3.ETH1**	10,249	1,172	7,215	809
**L1.2.1**	26,792	2,058	18,086	1,419
**L1.2.2**	26,567	2,035	5,484	448
**L7**	58,276	4,461	11,280	927

Supplemental Experimental Procedures

Correlation between dating analyses and known Ethiopia historical events

Based on the MTBC-70 model, the dominant Ethiopian sub-lineage L4.2 (15% of all 950 isolates based on spoligotype analysis [S1]) has a coalescent date consistent with its arrival in association with the major north-south human migration and origin of the Ethiosemitic languages around 3 thousand years ago [S3] (Table S2). L4.2 includes the "Ural" spoligotype family commonly found in the Pontic region north of the Black Sea and in Iran [S4], and a plausible scenario could involve infection of a Neolithic human population in the Levant by the L4.2 progenitor, followed by divergent north/south migrations. Sub-lineage L4.6 has an older coalescent point, corresponding to 20 thousand years ago in the MTBC-70 model. A potential link with patterns of human migration is provided by Eurasian mitochondrial DNA haplogroups U6 and M1 which were present in Africa during the Upper Paleolithic period prior to Neolithic expansion [S5]. L4.6 is currently restricted to Africa, and its common occurrence in Uganda, Cameroon and neighbouring countries suggests that it could have been disseminated across southern Africa in association with the Bantu expansion between 1000 BC and 500 AD. The remaining two sub-lineages of L4 observed in Ethiopia may represent recent colonial spread from Europe as evidenced, for example, by the global distribution of the spoligotype family known as "Latin American Mediterranean" (LAM) [S6]. Coalescent analysis of L3 suggests a similar pattern of multiple introductions into Ethiopia. Dating by the MTBC-70 model suggests that the major sub-lineage L3.ETH1 was established in Ethiopia prior to the Neolithic period. The geographic distribution of L3.ETH1, together with its early divergence from sub-lineages present in the Indian sub-continent, suggests an introduction into Ethiopia by ancient land-based human migrations. Coalescent analysis of other Ethiopian L3 isolates mapping to sub-lineages with a broader geographic distribution would be consistent with a more recent introduction through trade routes across the Indian Ocean.

Coalescent times estimated according to the MTBC-6 model suggest a much more recent origin and spread of *M. tuberculosis* in Ethiopia between the 15th and 19th centuries (Table S2). These dates are difficult to reconcile with major population migrations into Ethiopia, though historical records show continuous internal migrations with southward movement of Tigrean populations during ancient and medieval periods, northward migration of Oromos between the 16th and early 18th centuries, and southern movement of Amharas in the 19th century. Documentation of a recent expansion of L2 genotype in South Africa demonstrates that large-scale population movements are not a prerequisite for effective dissemination of a novel strain within a susceptible population, however, while the absence of an association of the West African slave trade with Lineages 5 and 6 shows that *M. tuberculosis* genotypes do not inevitably follow population movements. The predicted 16th century introduction of L4.2 into Ethiopia could reflect a period of increasing openness to European adventurers, with L4.2.ETH1 aligning with the timing of a Portuguese military expedition to support the resistance of Ethiopian Christians against Muslim incursions. The 12th century dating of L3.4

and L3.ETH1 would be consistent with the dissemination of these clades by Indian Ocean trade.

Supplemental References

S1. Firdessa, R., Berg, S., Hailu, E., Schelling, E., Gumi, B., Erenso, G., Gadisa, E., Kiros, T., Habtamu, M., Hussein, J., et al. (2013). Mycobacterial lineages causing pulmonary and extrapulmonary tuberculosis, Ethiopia. Emerg. Infect. Dis. *19*, 460–463.

S2. Demay, C., Liens, B., Burguiere, T., Hill, V., Couvin, D., Millet, J., Mokrousov, I., Sola, C., Zozio, T., and Rastogi, N. (2012). SITVITWEB--a publicly available international multimarker database for studying *Mycobacterium tuberculosis* genetic diversity and molecular epidemiology. Infect. Genet. Evol. *12*, 755–766.

S3. Pagani, L., Kivisild, T., Tarekegn, A., Ekong, R., Plaster, C., Romero, I. G., Ayub, Q., Mehdi, S. Q., Thomas, M. G., Luiselli, D., et al. (2012). Ethiopian genetic diversity reveals linguistic stratification and complex influences on the ethiopian gene pool. Am. J. Hum. Genet. *91*, 83–96.

S4. Mokrousov, I. (2011). The quiet and controversial: Ural family of *Mycobacterium tuberculosis*. Infect. Genet. Evol. *12*, 619-629.

S5. Pennarun, E., Kivisild, T., Metspalu, E., Metspalu, M., Reisberg, T., Moisan, J., Behar, D. M., Jones, S. C., and Villems, R. (2012). Divorcing the Late Upper Palaeolithic demographic histories of mtDNA haplogroups M1 and U6 in Africa. BMC Evol. Biol. *12*, 234.

S6. Mokrousov, I., Vyazovaya, A., and Narvskaya, O. (2014). *Mycobacterium tuberculosis* Latin American-Mediterranean family and its sublineages in the light of robust evolutionary markers. J. Bacteriol. *196*, 1833–1841.
